# pH-Responsive Dual Drug-Loaded Nanocarriers Based on Poly (2-Ethyl-2-Oxazoline) Modified Black Phosphorus Nanosheets for Cancer Chemo/Photothermal Therapy

**DOI:** 10.3389/fphar.2019.00270

**Published:** 2019-03-19

**Authors:** Nansha Gao, Chenyang Xing, Haifei Wang, Liwen Feng, Xiaowei Zeng, Lin Mei, Zhengchun Peng

**Affiliations:** ^1^Key Laboratory of Optoelectronic Devices and Systems of Ministry of Education, College of Optoelectronic Engineering, Shenzhen University, Shenzhen, China; ^2^School of Pharmaceutical Sciences (Shenzhen), Sun Yat-sen University, Guangzhou, China

**Keywords:** synergistic cancer therapy, black phosphorus, co-delivery, pH-responsive, charge reversal

## Abstract

Synergistic cancer therapy, such as those combining chemotherapeutic and photothermal methods, has stronger treatment effect than that of individual ones. However, it is challenging to efficiently deliver nanocarriers into tumor cells to elevate intracellular drug concentration. Herein, we developed an effective pH-responsive and dual drug co-delivery platform for combined chemo/photothermal therapy. An anticancer drug doxorubicin (DOX) was first loaded onto the surface of black phosphorus (BP). With poly(2-ethyl-2-oxazoline) (PEOz) ligand conjugated onto the polydopamine (PDA) coated BP nanosheets, targeted long circulation and cellular uptake *in vivo* was significantly improved. With another anticancer drug bortezomib (BTZ) loaded onto the surface of the nanocapsule, the platform can co-deliver two different drugs. The surface charge of the nanocapsule was reversed from negative to positive at the tumor extracellular pH (∼6.8), ionizing the tertiary amide groups along the PEOz chain, thus facilitating the cell internalization of the nanocarrier. The cytotoxicity therapeutic effect of this nanoplatform was further augmented under near-infrared laser irradiation. As such, our DOX-loaded BP@PDA-PEOz-BTZ platform is very promising to synergistic cancer therapy.

## Introduction

Black phosphorus (BP), as a novel 2D material, has attracted global attention owing to outstanding optoelectronic properties and wide applications (Han et al., 2017; Zhou et al., 2017). Bulk BP can be easily exfoliated into nanosheets (NSs) with different thicknesses (Churchill and Jarillo-Herrero, 2014; Yasaei et al., 2015). Compared with other 2D materials such as graphene and MoS_2_, BP has a larger specific surface area to adsorb large amounts of theranostic agents or antitumor drugs, thereby being potentially applicable to drug delivery (Tao et al., 2017). Unlike other 2D materials, the bandgap voltage of BP is related with its number of layers, ranging from 0.3 eV (a bulk value) to ∼2.0 eV (a monolayer value), (Liu et al., 2015) so it has absorptions in both UV and near-infrared (NIR) regions. Thus, BP NSs have unique optoelectronic performance (Shao et al., 2016) to work as either an excellent nano-optoelectronic device or an effective photothermal agent for photothermal therapy (PTT) due to high photothermal conversion efficiency and NIR extinction coefficient (Guo et al., 2015; Sun et al., 2015, 2016; Xing et al., 2017, 2018b). Therefore, antitumor drug doxorubicin (DOX) or paclitaxel are often loaded onto BP NSs to fabricate multifunctional drug delivery systems for synergistic cancer chemotherapy/PTT.

Nevertheless, the application of BP is greatly hindered, because it is prone to degradation into P*x*O*y* in the air and aqueous solutions (Liu et al., 2014; Doganov et al., 2015). To this end, researchers have endeavored to stabilize BP NSs through surface modification strategies such as ligand surface coordination (Zhao et al., 2016; Guo et al., 2017), covalent aryl diazonium functionalization (Ryder et al., 2016) and capping layer protection (Wood et al., 2014), which, however, are unsuitable for human drug delivery for either introducing toxic substances or weakening photothermal outcomes. As a surface-adherent biomimetic material formed through the oxidative self-polymerization of dopamine under alkaline conditions, polydopamine (PDA) is inspired by marine mussel and has been widely used a coating on nanomaterial surfaces owing to high biodegradability, biocompatibility and pH responsiveness at low pH values (Lee et al., 2007; Liu et al., 2013; Cheng et al., 2017a). We have previously elevated the stability of BP NSs in aqueous solution by simply modifying their surface with PDA safely and effectively, without attenuating the photothermal effects (Gao et al., 2018), based on the photothermal conversion efficiency of PDA (Liu et al., 2013; Cheng et al., 2017b; Peng et al., 2018).

Besides, BP NSs can be readily phagocytosed and cleared by the mononuclear phagocytic system (MPS) after being injected *in vivo*. Conventionally, the *in vivo* circulation of nanocarriers is prolonged through surface modification with hydrophilic or zwitterionic polymers among which polyethylene glycol (PEG) is most investigated and utilized due to excellent biocompatibility and hydrophilicity, especially for the polymers based drug delivery (Chen et al., 2017a; Feng et al., 2017; Guo et al., 2018; Wang et al., 2018). This technique is also known as PEGylation (Zeng et al., 2013; Cheng et al., 2017c; Nie et al., 2017; Zhao et al., 2017; Xiao et al., 2018; Tang et al., 2019). However, it suffers from the following issues. First, PEGylated therapeutic agents, after being administered repeatedly, cannot fully escape from being phagocytosed by cells in MPS, and the immunogenicity is bound to induce obvious humoral immune response. Therefore, they are recognized and removed by the immune system. Second, PEGylated liposome and nanoparticles can be immunoreactive to induce an accelerated blood clearance (ABC) phenomenon (Wang et al., 2007; Tsai et al., 2018). Third, the *in vivo* stability of PEG is affected, because its polyether main chain easily undergoes oxidative degradation. Burt et al. (1999) found the cleared PEG and PDLLA fragments of (ethylene glycol)-block-poly(D,L-lactide) (PEG-b-PDLLA) micelles in mouse urine. Furthermore, it is difficult to conjugate the surface of PEGylated nanocarriers with functional ligands because PEG has limited reactive groups, thus requiring an alternative technique to stabilize BP NSs for *in vivo* biomedical applications (Wu et al., 2018). In recent years, poly(2-ethyl-2-oxazoline) (PEOz) has been verified as a high-molecular weight, long-chain polymer with high water solubility, flexibility and biocompatibility, and approved by the United States Food and Drug Administration. Meanwhile, PEOz is capable of long circulation *in vivo*, inhibiting protein adsorption and decreasing blood clearance, as a qualified substitute for PEG. Compared with PEG, PEOz has a more stable main chain which facilitates the introduction of various active groups and provides a chemical basis for further linking to target molecules. Notably, with unique tertiary amide groups in the main chain, PEOz has a similar p*K*a to that of physiological pH, which can be adjusted by varying the molecular weight. At pH lower than its pKa, PEOz is reversed from negatively to positively charged through ionization of tertiary amide groups along the PEOz chain. As a result, PEOz-modified drug nanodelivery system can be enriched and charge-reversed in the weakly acidic environment of tumor tissue (pH ∼6.8), allowing endocytosis and pH-responsive drug release after being induced by the low pH (∼5.0) of endosomes and lysosomes. Finally, the drug release rate is controllable and the tumor-targeting ability is improved, managing to enhance the antitumor activities and to reduce the side effect simultaneously (Gao et al., 2015a,b,c; Zhao et al., 2015; Wang et al., 2017).

As a broad-spectrum antitumor agent, DOX can be intercalated into DNA of tumor cells to suppress nucleic acid synthesis, exerting therapeutic effects on acute leukemia and a variety of solid tumors. Nevertheless, it is highly cytotoxic and easily degradable, without selectivity or specificity (Chen et al., 2017b; Xing et al., 2018a; Zhang et al., 2018). Bortezomib (BTZ), on the other hand, is a common clinical antitumor agent applicable to patients with multiple myeloma, which can inhibit tumor cell growth by binding the threonine residues of active sites of several proteases and induce apoptosis mainly via the mitochondrial pathway. However, the therapeutic effects of BTZ on many types of solid tumors are limited, because it is non-specifically bound to normal cell proteins and can thus be rapidly cleared by the blood, accompanied by dose-related cytotoxicity (Su et al., 2011). Consequently, we herein designed a nanodrug BP-DOX@PDA-PEOz-BTZ carrying DOX and BTZ simultaneously for the chemo/photothermal combination therapy of breast cancer. The PDA layer enhanced the system stability before reaching the tumor site, and maintained the remarkable photothermal effects for subsequent modification. Afterward, PEG was replaced by PEOz to prolong *in vivo* drug circulation and to increase cellular uptake. In the meantime, a pH-targeted controlled release trigger system was constructed to remedy the deficiency of chemical drugs in solid tumor therapy, and to further boost the antitumor effects relying on high photothermal conversion efficiency. This system is conducive to chemo/photothermal combination therapy by not only raising the drug loading content, cellular uptake and pH-responsive release rate, but also exhibiting high photothermal activity against tumor cells.

## Materials and Methods

### Materials

Bulk BP was purchased from Smart-Elements (Austria) and stored in a 4°C refrigerator. Dopamine hydrochloride was bought from Sigma-Aldrich (St. Louis, MO, United States). H_2_N-PEG was obtained from Shanghai Yare Biotech, Inc. (China). H_2_N-PEOz was bought from Xi’an Ruixi Biological Technology Co., Ltd. (China). The molecular weights (M*_w_*) of PEG and PEOz were both 2 kDa. DOX was purchased from Dalian Meilun Biology Technology Co., Ltd. (China). BTZ was obtained from Beijing Zhongshuo Pharmaceutical Technology Development Co., Ltd. (China). Other analytical-grade reagents and solvents were used as received. HyPure Molecular Biology Grade Water (Hyclone^TM^) was used to prepare all solutions.

### Preparation of BP NSs

Black phosphorus NSs were fabricated by simply exfoliating the corresponding bulk BP sample in liquid. Briefly, 20 mg of BP was dispersed in 20 mL of 1-methyl-2-pyrrolidinone (NMP) which was argon-bubbled to decrease oxidation by eliminating dissolved oxygen molecules during exfoliation. The mixture was thereafter sonicated in an ice water bath for 8 h (amplifier: 25%, on/off cycle: 5 s/5 s). The low system temperature was kept by the ice water bath. Afterward, unexfoliated bulk BP was removed by centrifuging the brown dispersion at 2,000 rpm for 10 min, and the supernatant containing BP NSs was carefully collected for further use.

### DOX Loading Onto BP NS Surface

Doxorubicin (2 mg) was mixed with 2 mL of 1 mg mL^-1^ BP NSs solution in water, and the solution pH was adjusted to 8.5 with sodium hydroxide. After being stirred vigorously in dark overnight, the obtained DOX-loaded BP (BP-DOX) NSs were collected by centrifugation and washed by water. The LC (%) of DOX were calculated using the following equation.

$$\begin{array}{c} \text{Drug LC}(\%)\;=\;\frac{weight\;of\;drug\;in\;the\;nanoparticles}{weight\;of\;nanoparticles}\times 100\% \end{array}$$

### PDA Coating on BP NS Surface

BP-DOX NSs were dispersed in 2 mL HyPure Molecular Biology Grade Water at 1 mg mL^-1^. Then pH was adjusted to 8.5 by adding sodium hydroxide, and the solution was added 10 μL dopamine hydrochloride (100 mg mL^-1^) and stirred for 2.5 h in dark at room temperature. Finally, BP-DOX@PDA particles were collected by 10 min of centrifugation at 12,000 rpm and washed by deionized water.

### Conjugation of H_2_N-PEOz or H_2_N-PEG Onto BP-DOX@PDA Surface

PDA-coated NSs (2 mg) were first resuspended in 2 mL of HyPure Molecular Biology Grade Water with pH adjusted to 8.5 by an appropriate amount of sodium hydroxide. After 2 mg of H_2_N-PEOz was added into the BP@PDA suspension, the mixture was vigorously stirred for 3 h in dark at room temperature. Then the H_2_N-PEOz-modified NPs (BP-DOX@PDA-PEOz) were purified by 10 min of centrifugation at 12,000 rpm and washed by deionized water. With a similar procedure, BP-DOX@PDA-PEG was fabricated by using H_2_N-PEG instead of H_2_N-PEOz.

### BTZ Loading Onto PDA-Coated NPs

In brief, 50 mg of BP-DOX@PDA-PEOz NPs were suspended at 1 mg mL^-1^ in deionized water with pH adjusted to 8.5 by sodium hydroxide, and 6 mg of BTZ powders were dispersed in 200 μl of DMSO. Under stirring, the latter solution was then dropwise added to the former solution. Afterward, the mixture was stirred overnight and centrifuged with the same process as that described above. After 48 h of lyophilization, the product was referred to as BP-DOX@PDA-PEOz-BTZ.

### Characterizations of BP NSs

Transmission electron microscopy (TEM) images were acquired by Tecnai G2 F30 transmission electron microscope (FEI, Hillsboro, OR, United States). BP NSs were observed after being dropped onto a copper grid-coated carbon membrane and air-dried. Fourier transform infrared (FTIR) spectra were recorded with Nicolet iS 50 spectrometer (Thermo Scientific, United States). Raman spectra were recorded at room temperature by LabRAM HR800 high-resolution confocal Raman microscope (HORIBA, United States). X-ray photoelectron spectroscopy was performed with Axis HSi X-ray photoelectron spectroscope (Kratos Ltd., United Kingdom) employing Al Kα radiation (150 W, 1486.6 eV photons) as the excitation source. Zeta potential and size were measured by Malvern Mastersizer 2000 particle size analyzer (Zetasizer Nano ZS90, Malvern Instruments Ltd., United Kingdom). All measurements were conducted three times independently and averaged.

### Photothermal Effects of Different BP NSs

The aqueous solutions (0.1 mg mL^-1^) of different NSs (BP NSs, BP@PDA, BP@PDA-PEOz) were added into microfuge tubes. With the same process, water was utilized as control. The middle of each solution was irradiated at 808 nm with KS-810F-8000 fixed fiber-coupled continuous semiconductor diode laser (Kai Site Electronic Technology Co., Ltd., Xi’an, China) at the power density of 1.0 W cm^-2^. To evaluate the effects of concentration changes, BP@PDA-PEOz solutions with various concentrations of NSs (10–200 μg mL^-1^) were tested by recording the temperature changes under the above-mentioned irradiation. Also, BP@PDA-PEOz solution (100 μg mL^-1^) was tested at various power densities (0.5–2.0 W cm^-2^) to monitor the temperature changes. Ti450 IR thermal imaging camera (Fluke, United States) was used for temperature recording.

### pH and Photothermal-Induced Drug Release Profiles

To evaluate the DOX release profile of BP-DOX@PDA-PEOz-BTZ NSs, 5 mg of NSs were resuspended in 1 mL of phosphate-buffered saline (PBS, pH = 5.0, 6.8 or 7.4, containing 0.1% w/v Tween 80). Subsequently, the dispersion was transferred into a dialysis membrane bag [MWCO = 3500, Sangon Biotech (Shanghai) Co., Ltd., China] that was then incubated in 10 mL of PBS at pH 5.0, 6.8 or 7.4 in an orbital water bath and shaken at 37°C. At dedicated time points, 0.5 mL of the solution outside was collected to detect the amount of released DOX with UV–vis spectrometer at 490 nm, which was supplemented by 0.5 mL of fresh PBS. Under identical conditions, photothermal-triggered drug release was tested at pH 5.0 with 6 min of 808 nm laser irradiation at the power density of 1.0 W cm^-2^. *In vitro* BTZ release and photothermal-triggered drug release from BP-DOX@PDA-PEOz-BTZ NSs were detected by LC 1200 HPLC system (Nie et al., 2017) (Agilent Technologies, Santa Clara, CA, United States). Compounds were separated by a reverse-phase C18 column (5 μm, 150 × 4.6 mm; Agilent Technologies, Santa Clara, CA, United States) using a mobile phase comprising deionized water and acetonitrile (20/80 for BTZ, v/v). The flow rate was 1.0 mL/min and the injection volume was 20 μl. BTZ amount was measured by UV–vis spectroscopy at 270 nm. Finally, the accumulative release versus time profiles of BTZ and DOX were plotted.

### Cell Culture Assays

Breast cancer cell line MCF-7 was chosen to study the endocytic behaviors of the above NPs. The cells were incubated in Dulbecco’s modified Eagle’s medium (DMEM) (Life Technologies, Carlsbad, CA, United States) containing 100 μg mL^-1^ streptomycin, 100 U mL^-1^ penicillin and 10% (v/v) fetal bovine serum at 37°C with 5% CO_2_.

### Cellular Uptake of NPs

MCF-7 cells were inoculated into 20 mm glass-bottomed Petri dishes and thereafter incubated for 24 h. BP@PDA-PEG and BP@PDA-PEOz loading 10 μg mL^-1^ DOX were added into the wells simultaneously at pH 6.8 or 7.4, followed by 4 h of incubation at 37°C. The cells were washed three times by PBS, and observed with Fluoview FV-1000 confocal laser scanning microscope (Olympus, Tokyo, Japan) at 488 and 590 nm as the excitation and emission wavelengths, respectively.

### Cytotoxicity Assay

The cytotoxicities of BP@PDA-PEG and BP@PDA-PEOz were determined by the MTT assay. MCF-7 cells were seeded into a 96-well plate at the density of 5 × 10^3^ and thereafter incubated for 24 h. Afterward, the medium was replaced by 100 μL of fresh medium containing different concentrations of BP@PDA-PEG or BP@PDA-PEOz (10, 25, 100 μg mL^-1^), and the cells were further incubated for 48 h. Then the medium was replaced by MTT solution in DMEM (5 mg mL^-1^, 100 μL), followed by another 4 h of incubation. The supernatant was removed from each well into which 100 μL of DMSO was then added to dissolve the formed formazan crystals. The absorption of each well was detected at 570 nm by Model 680 microplate reader (Bio-Rad, United Kingdom).

### *In vitro* PTT Study

MCF-7 cells were inoculated into a 96-well plate and incubated for 24 h. After the medium in each well was refreshed, the cells were incubated with various concentrations (10, 25, 50 μg mL^-1^) of BP@PDA-PEG and BP@PDA-PEOz at 37°C for 4 h, irradiated for 10 min by 808 nm laser (1.0 W cm^-2^) and incubated again for 12 h. The cell viability was assessed by the MTT assay.

### *In vitro* Cell Viabilities at Different pH Values

MCF-7 cells were incubated overnight after being inoculated into 96-well plates. Subsequently, they were incubated by BP-DOX@PDA-PEG/BP-DOX@PDA-PEOz/BP-DOX@PDA-PEOz-BTZ with equivalent DOX concentrations at pH 6.8 or 7.4. The MTT solution was added after 48 h, and the cell viability was also detected with the microplate reader at 570 nm. The viability of untreated cells was set at 100%.

### *In vitro* Combined Antitumor Therapy

MCF-7 cells were inoculated into a 96-well plate at 1 × 10^4^/well, and incubated overnight. The adherent cells were treated with DOX, BTZ, DOX + BTZ (1:1), BP-DOX@PDA-PEG, BP-DOX@PDA-PEOz, BP-DOX@PDA-PEOz-BTZ, BP-DOX@PDA-PEG + NIR, BP-DOX@PDA-PEOz + NIR, and BP-DOX@PDA-PEOz-BTZ + NIR (808 nm laser irradiation, 1.0 W cm^-2^) at 0.25, 1, 2.5, and 5 μg mL^-1^ equivalent DOX concentrations for 24 and 48 h. After treatment with the NSs for 24 or 48 h in the presence or absence of NIR laser irradiation, the cell viability was tested by the MTT assay. The optical density of each well at 570 nm was measured by the microplate reader. The viability of untreated cells was set at 100%, and the absorbance of the control group was zero.

### *In vivo* IR Thermal Imaging

All animal experiments have been approved by the Administrative Committee on Animal Research in Sun Yat-sen University, and *in vivo* experiments were conducted according to corresponding guidelines. Female severe combined immunodeficient (SCID) mice aged 5–6 weeks old were provided by the Guangdong Medical Laboratory Animal Center and given free access to water and food. Each mouse was subcutaneously injected with 100 μL PBS suspension of MCF-7 cells (∼2 × 10^6^) on the dorsal side to induce tumors. Every 2 days, the length and width of tumors were measured with a digital vernier caliper to estimate the volumes using the formula: 0.5 × (length) × (width)^2^. SCID mice bearing MCF-7 tumors were employed as the animal model. IR thermal imaging was carried out when the tumor volumes reached approximately 180 mm^3^. The mice were thereafter intravenously injected with PBS, BP@PDA-PEG, BP@PDA-PEOz or BP@PDA-PEOz-BTZ, and the BP dose is 5 mg/kg in 100 μl PBS. Twenty-four hours later, the tumor sites were irradiated for 5 min by 808 nm laser (1.5 W cm^-2^). IR thermographic maps and temperature changes were recorded with the IR thermal imaging camera.

### Statistical Analysis

Unless otherwise stated, all experiments were performed at least in triplicate, and the data were represented as mean ± standard deviation. Statistical analysis was carried out by using SPSS 22.0 software for one-way analysis of variance and subsequent Bonferroni test. ^∗^*P* < 0.05 and ^∗∗^*p* < 0.01 were considered statistically significant and extremely statistically significant, respectively.

## Results and Discussion

### Morphology and Characterizations

**FIGURE 1:**
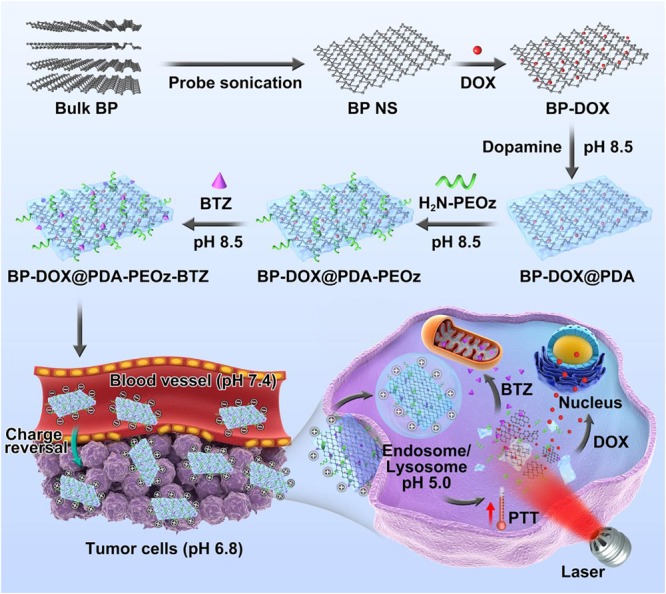
Schematic representation of dual-drug-loaded BP-DOX@PDA-PEOz-BTZ and summary of the endocytosis pathways and chemo-photothermal synergistic therapy of cancer.

The entire synthesis procedure of the BP-based drug delivery platform, including DOX loading, PDA coating, PEOz conjugation and BTZ loading, is shown in Figure 1. According to the modified liquid exfoliation technique reported by our group previously (Tao et al., 2017), BP NSs were prepared from bulk BP in NMP. Then DOX was absorbed onto the corrugated surface of BP NSs by non-covalent bonding such as Van der Waals force and electrostatic attraction. The DOX loading capacity of BP can reach over 300% in a weakly alkaline condition (Gao et al., 2018). In a weakly alkaline solution, dopamine monomer underwent oxidative self-polymerization into PDA, ultimately adhering to the surface of NPs (Lee et al., 2007; Postma et al., 2009; Hong et al., 2012). PDA coating enhanced the system stability in physiological medium, probably as a universal bond between NSs and ligand by being reactive with thiol and amine groups. The terminal amine group of NH_2_-PEOz conjugated to NSs coated with PDA through a simple Michael addition reaction. The oxidative self-polymerization mechanism of dopamine and the conjugation mechanism between H_2_N-PEOz and PDA coating were shown as Supplementary Figure S1A. As a long-chain molecule, PEOz was introduced into the system as a substitute for PEG to maintain long-term circulation, to attenuate the ABC phenomenon and to allow pH-responsive drug release. In alkaline solutions, the boronic acid active site in BTZ reacted with catechol in PDA, which may suppress the activity of BTZ and thus decrease non-specific cellular drug endocytosis. Additionally, BTZ was released under acidic conditions, possibly also facilitating drug release at the tumor site and selectively augmenting antitumor activity (Supplementary Figure S1B) (Su et al., 2011). In short, DOX-loaded BP@PDA-PEOz-BTZ may be applicable to synergistic chemotherapy/PTT by prolonging *in vivo* circulation as well as elevating cellular uptake efficiency, pH responsiveness and dual drug loading capacity.

**FIGURE 2:**
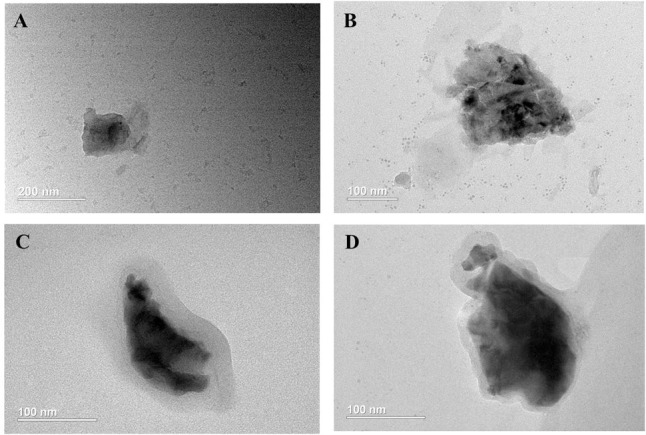
Characterization of BP NSs. TEM images of **(A)** BP NSs; **(B)** BP@PDA NSs; **(C)** BP@PDA-PEOz; **(D)** BP@PDA-PEOz-BTZ.

TEM images (Figure 2A–D) exhibit that BP NSs have sheet-like morphology, and bare BP and modified BP NSs have the lateral sizes of approximately 200–250 nm, being consistent with the results of dynamic light scattering analysis. After PDA and PEOz coating, the NS surface became rough and slightly thickened.

**FIGURE 3:**
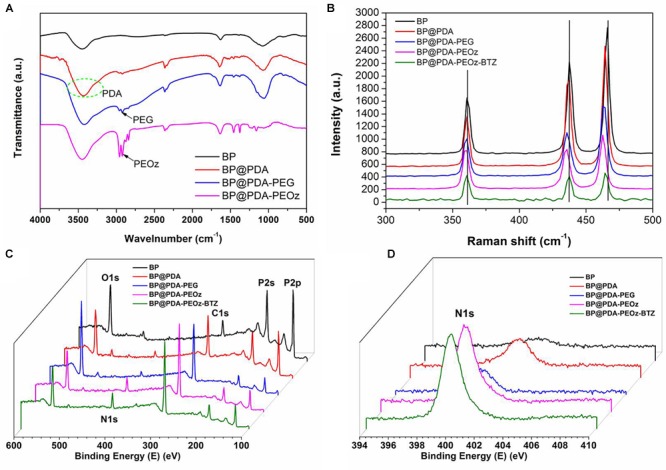
**(A)** FTIR spectra of BP NSs, BP@PDA NSs, BP@PDA-PEG and BP@PDA-PEOz. **(B)** Raman spectra of BP NSs, BP@PDA NSs, BP@PDA-PEG, BP@PDA-PEOz and BP@PDA-PEOz-BTZ; XPS spectra of BP NSs, BP@PDA NSs, BP@PDA-PEG, BP@PDA-PEOz, and BP@PDA-PEOz-BTZ. **(C)** Survey spectrum and **(D)** N1s spectrum.

Figure 3A presents the FTIR spectra of BP NSs, BP@PDA, BP@PDA-PEG, and BP@PDA-PEOz. The adsorption peak at ∼1,625 cm^-1^ represents P=O stretching vibration (Shen et al., 2015). After PDA coating, a broad and intense band between 3,150 and 3,600 cm^-1^ appears, corresponding to N-H/O-H stretching vibration. The peak at ∼1,500 cm^-1^ can be assigned to the bending vibrations of benzene ring and N-H in PDA. In the spectrum of BP@PDA-PEG, the peak at about 2,900 cm^-1^ represents C-H stretching vibration, suggesting that PEG had been successfully modified. The peak at 1,640 cm^-1^ is related to the C=O stretching vibration of imide bond in PEOz (Qiu et al., 2013). Moreover, the spectrum of BP@PDA-PEOz shows a peak at ∼2,900 cm^-1^ corresponding to C-H stretching vibration (Yu et al., 2014), indicating successful modification of PEOz.

The structures of PDA- and PEOz-modified BP NSs were studied by Raman spectroscopy (Figure 3B). In the spectrum of bare BP, there are three obvious peaks at ∼360.7, 437.5, and 466.1 cm^-1^ which correspond to the A^1^_g_, B_2g_, and A^2^_g_ modes of BP, respectively. The peaks of modified NSs (BP@PDA, BP@PDA-PEG, BP@PDA-PEOz, BP@PDA-PEOz-BTZ) shift toward lower wavenumber slightly, which can be ascribed to the mild ultrastructural changes after PDA coating and further modification, demonstrating successful modification of PDA and PEOz. The surface modification of NSs loading BTZ was confirmed by X-ray photoelectron spectroscopy (Figure 3C,D and Supplementary Figure S2). As evidenced by the intensity increase of nitrogen peak (N1s) at 399.49 eV (Figure 3D), both PDA coating and PEOz/BTZ loading were successful. The P2p peak (129.6 eV) intensities of bare BP, BP@PDA, BP@PDA-PEG, and BP@PDA-PEOz gradually drop (Supplementary Figure S2A) because of the coverage of P element. Collectively, corresponding compounds had indeed been successfully modified (Wang et al., 2015).

The results of dynamic light scattering are listed in Table 1. The hydrodynamic sizes increased slightly owing to layer-by-layer modification with PDA, PEG or PEOz, being in accordance with the TEM results. Additionally, the appropriate size and narrow size distribution may be beneficial to NSs accumulation in tumors through the EPR effect (Torchilin, 2011). Besides, the zeta potential of bare BP NSs was -18.9 mV, while that after surface modification with PDA became -16.6 mV, probably because phenolic hydroxyl groups on the PDA layer were deprotonated at neutral pH (Cheng et al., 2017c; Nie et al., 2017). After NH_2_-PEOz and NH_2_-PEG modification, the zeta potentials of BP@PDA-PEOz and BP@PDA-PEG in deionized water were measured to be -10.5 mV and -13.2 mV, respectively.

**Table 1:** Characterization of BP-based NSs in deionized water.

Sample	Size (nm)	PDI	ZP (mV)
BP	217.1 ± 15.3	0.145	–18.9 ± 3.2
BP@PDA	224.5 ± 18.1	0.150	–16.6 ± 2.7
BP@PDA-PEG	232.7 ± 20.2	0.174	–13.2 ± 1.1
BP@PDA-PEOz	236.3 ± 19.4	0.163	–10.5 ± 1.8
BP@PDA-PEOz-BTZ	239.2 ± 21.8	0.127	–9.3 ± 1.5
BP-DOX@PDA-PEG	238.4 ± 25.6	0.161	–8.1 ± 1.0
BP-DOX@PDA-PEOz	241.5 ± 23.1	0.152	–6.4 ± 0.7
BP-DOX@PDA-PEOz-BTZ	248.6 ± 22.0	0.133	–4.9 ± 0.5

*PDI, polydispersity index; ZP, zeta potential, *n* = 3.*

### Effects of pH on Size and Zeta Potential

**FIGURE 4:**
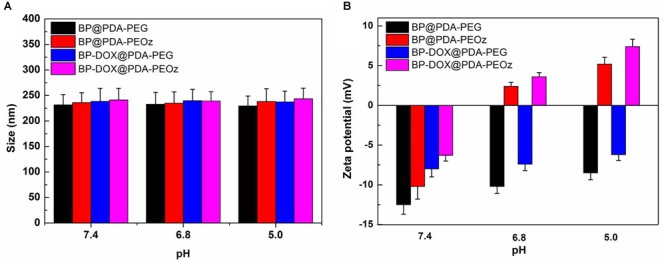
**(A)** Variations of the size for BP@PDA-PEG, BP@PDA-PEOz, BP-DOX@PDA-PEG, BP-DOX@PDA-PEOz in water solution with different pH of 7.4, 6.8 and 5.0 values at 37 °C (*n* = 3). **(B)** Zeta potential of the BP@PDA-PEG, BP@PDA-PEOz, BP-DOX@PDA-PEG, BP-DOX@PDA-PEOz in different pH of 7.4, 6.8, and 5.0 (*n* = 3).

To further evaluate the influence of pH, drug-free and DOX-loaded BP@PDA-PEG/BP@PDA-PEOz NSs (1 mg mL^-1^) were exposed in 10 mM PBS at pH 5.0, 6.8 or 7.4 and sonicated for 30 min at 37°C before their sizes and zeta potentials were measured. As shown in Figure 4A, the sizes of all NSs barely change with reducing pH. BP@PDA-PEOz became slightly negatively charged under physiological conditions (pH 7.4), with the zeta potential of -10.2 mV (Figure 4B). As pH decreased from 7.4 to 5.0, the surface charge was reversed from negative to positive, and the zeta potential rose to 2.4 mV at pH 6.8 and 5.2 mV at pH 5.0. The charge reversal can be attributed to ionization of the amide groups from PEOz in the outer layer, inducing partial charge neutralization. Taken together, the surface charge of BP@PDA-PEOz and BP-DOX@PDA-PEOz are positive while the pH values decreased from 7.4 to 6.8 and 5.0.

### *In vitro* Photothermal Effects

**FIGURE 5:**
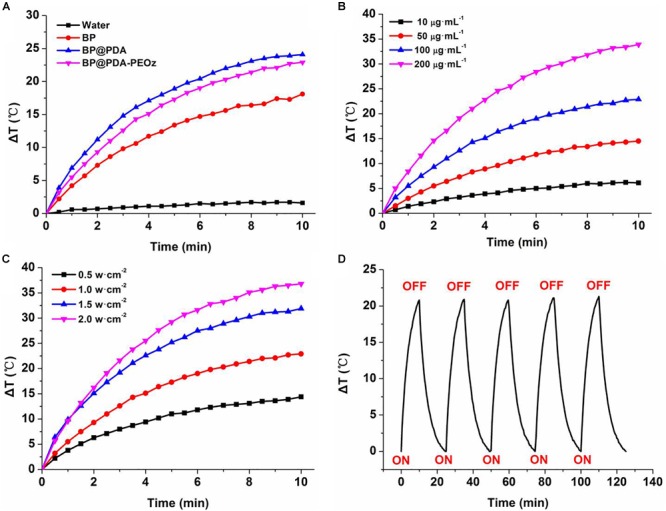
**(A)** Photothermal heating curves of pure water, BP, BP@PDA, and BP@PDA-PEOz solution under 808 nm laser irradiation (1.0 W/cm^-2^) for 10 min. **(B)** Photothermal heating curves of the BP@PDA-PEOz solution with different concentrations. **(C)** Photothermal heating curves of the BP@PDA-PEOz solution under various power intensities. **(D)** Heating of a suspension of the BP@PDA-PEOz in water for five laser on/off cycles with an 808 nm NIR laser at power density of 1.0 W/cm^-2^.

To clarify the photothermal performance of the prepared co-delivery platform, the temperature variations were tested under 808 nm laser irradiation for 10 min. As displayed in Figure 5A, the temperatures of bare BP NSs, BP@PDA and BP@PDA-PEOz solutions (0.1 mg mL^-1^) all soar compared with that of distilled water under identical irradiation conditions. The photothermal efficiency of BP@PDA (Δ*T* = 24.1°C) exceeded that of bare BP (Δ*T* = 18.1°C), and the temperature of BP@PDA-PEOz was elevated by 22.9°C. PDA coating may be responsible for the augmented photothermal response of BP@PDA, accompanied by considerable photothermal conversion efficiency. Furthermore, the photothermal properties of PDA-coated BP NSs were both concentration- and laser power-dependent (Figure 5B,C). After five cycles of NIR laser irradiation, the temperature no longer changed evidently (Figure 5D), so the sample was highly photostable. Moreover, the photostability of BP@PDA-PEOz NSs surpassed that bare BP NSs, without obviously losing photothermal conversion efficiency in 1 week (Supplementary Figure S3). Hence, PDA coating boosted the photothermal performance of BP NSs, and rendered them suitable for PTT due to high photostability and photothermal conversion efficiency.

### *In vitro* pH- and Photo-Responsive Drug Release Profiles

**FIGURE 6:**
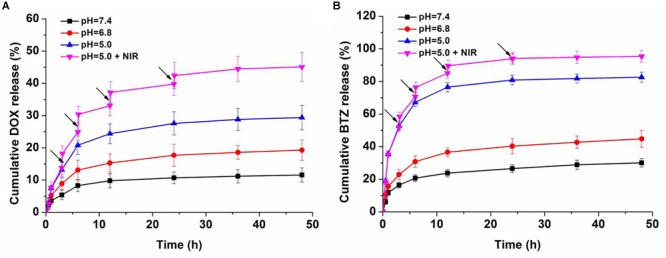
*In vitro* **(A)** DOX and **(B)** BTZ release profile of dual-drug-loaded BP-DOX@PDA-PEOz-BTZ in release medium with different pH values of 7.4, 6.8, and 5.0, the arrows show NIR irradiation for 0.1 h.

The sustained and controlled DOX release profiles of BP-DOX@PDA-PEOz-BTZ were tested at pH 7.4 for simulating normal physiological microenvironment, pH 6.8 for simulating tumor extracellular microenvironment and pH 5.0 for simulating the acidic microenvironment of tumor endosome/lysosome, in the presence or absence of NIR laser irradiation. At pH 5.0, nearly 30% of DOX was released from BP-DOX@PDA-PEOz-BTZ within 48 h, whereas only 11% of DOX was released at pH 7.4 (Figure 6A), which may be ascribed to the pH sensitivity of PEOz coating after tertiary amide groups along the PEOz chain were ionized at a pH value lower than its pKa (Nie et al., 2017; Wang et al., 2017). The positive charges on the nitrogen atoms of PEOz main chains may result in electrostatic repulsion, which loosened the outer shell in the slightly acidic tumor cell microenvironment (Wang et al., 2017) and accelerated the release of inner hydrophobic anticancer drugs into tumor tissues while reducing that into the normal blood circulation. As a result, the anticancer effect was increased, and the side effects of common anticancer drugs were relieved (Gao et al., 2015a; Zhao et al., 2015; Wang et al., 2017).

Moreover, the photo-responsive drug release behaviors were studied. After 808 nm laser irradiation (1.0 W cm^-2^, 6 min for each pulse), the temperature of BP-DOX@PDA-PEOz-BTZ increased gradually at pH 5.0, which significantly raised the cumulative DOX release amount, reaching above 40% after irradiation four times. Presumably, the PDA layer decomposed and released the loaded drug after NIR laser irradiation. In the meantime, BP decomposed gradually due to NIR exposure, further inducing drug release (Zeng et al., 2018).

Furthermore, we investigated the drug release behaviors of BTZ from BP-DOX@PDA-PEOz-BTZ NSs at different pH values with or without IR irradiation (Figure 6B). Merely approximately 25% of BTZ was released at pH 7.4 after 24 h. At pH 6.8 and 5.0, BTZ release was significantly accelerated. After NIR laser irradiation for 6 min, about 95% of BTZ was released from DOX-loaded BP@PDA-PEOz-BTZ within 48 h at pH 5.0. In other words, the pH sensitivity of catechol-BTZ bond contributed to BTZ accumulation at tumor sites, so the treatment outcomes were improved. The drug BTZ was loaded onto the surface of nanoplatform through the reversible covalent bond between catechol and phenylboronic acid. However, the drug DOX was absorbed onto the corrugated surface of BP NSs by non-covalent bond. And the DOX-loaded BP NSs was covered by the PDA. That is, the drug DOX is at the interior of the nanoplatform and BTZ is at exterior. Therefore, the release rate for DOX is in general much lower compared to the release rate of BTZ. Similar results were reported by our previous research (Nie et al., 2017). This release behavior of BTZ highlights the pH sensitivity of the catechol-BTZ bond contributing to the accumulation of BTZ at the tumor sites. The release pattern was very important for better tumor killing effect, as it reduces the drugs leakage during the circulation in blood and increases the drugs enrichment in tumor sites or endosomes. Overall, the pH-sensitive drug release triggered by NIR laser irradiation markedly enhanced the antitumor efficacy and minimized the side effect.

### Cellular Uptake of NSs

**FIGURE 7:**
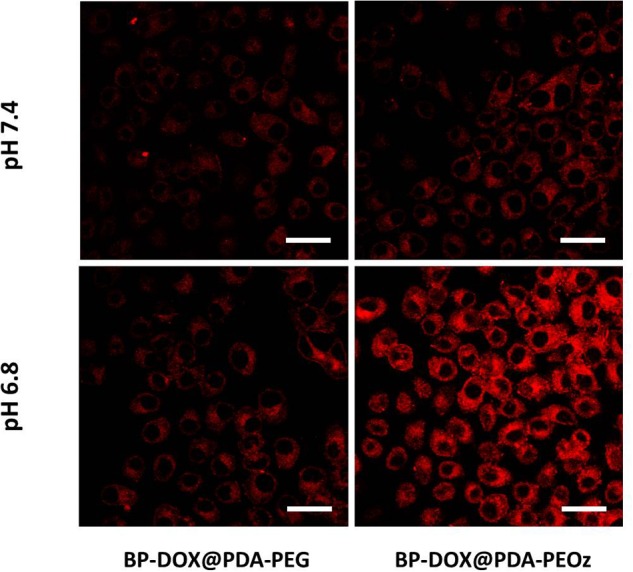
Confocal laser scanning microscopy images of MCF-7 cells incubated with the solution of BP-DOX@PDA-PEG and BP-DOX@PDA-PEOz at different pH values after 4 h. DOX concentration: 10 μg mL^-1^. Scale bar is 20 μm.

The uptake of BP-DOX@PDA-PEG and BP-DOX@PDA-PEOz NSs by MCF-7 cells in the weakly acidic tumor microenvironment (pH 7.4 and 6.8) was observed by confocal laser scanning microscopy. After treatment with different NSs for 4 h, the intracellular fluorescent intensities of BP-DOX@PDA-PEG NSs at pH 6.8 and 7.4 were similar, but the intensity of BP-DOX@PDA-PEOz NSs at pH 6.8 significantly exceeded that at pH 7.4 (Figure 7). The results validated the hypothesis that PEOz promoted the cellular uptake of DOX compared with PEGylated copolymer did in the mildly acidic endosomal/lysosomal and tumor extracellular environment, which can be attributed to the charge reversal of PEOz after tertiary amide groups along the PEOz chain were ionized (Cheng et al., 2017b; Wang et al., 2017; Zeng et al., 2017).

### Cell Viability

The *in vitro* cytotoxicities of drug-free BP@PDA-PEG and BP@PDA-PEOz as well as DOX-loaded BP@PDA-PEG, BP@PDA-PEOz and BP@PDA-PEOz-BTZ NSs were evaluated by the MTT assay. Drug-free BP@PDA-PEG and BP@PDA-PEOz NSs were also tested to eliminate the potential toxic characteristics of drug delivery capsule. Given that all drug-free BP-based NSs displayed negligible cytotoxicities against MCF-7 cells (Supplementary Figure S4), they were highly biocompatible.

**FIGURE 8:**
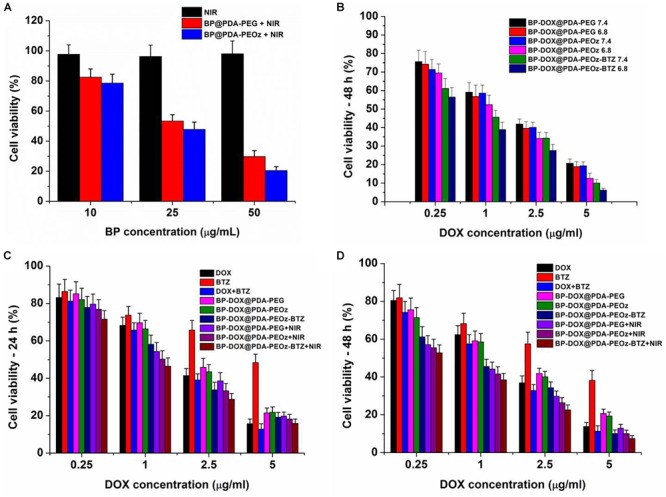
Viability of MCF-7 cells cultured with **(A)** fresh culture medium, BP@PDA-PEG, BP@PDA-PEOz under NIR irradiation for 10 min. **(B)** Viability of MCF-7 cells cultured with BP-DOX@PDA-PEG, BP-DOX@PDA-PEOz, and BP-DOX@PDA-PEOz-BTZ in culture medium of different pH 7.4 or 6.8. Viability of MCF-7 cells cultured with DOX-loaded nanoformulations in comparison with DOX, BTZ, DOX + BTZ (1:1) and DOX-loaded nanosheets of BP-DOX@PDA-PEG, BP-DOX@PDA-PEOz, BP-DOX@PDA-PEOz-BTZ, BP-DOX@PDA-PEG + NIR, BP-DOX@PDA-PEOz + NIR, and BP-DOX@PDA-PEOz-BTZ + NIR at the same DOX dose: **(C)** 24 h, **(D)** 48 h.

The photothermal cytotoxicities of different NSs were also evaluated by the MTT assay. Cell growth was barely affected by individual NIR laser irradiation, whereas BP@PDA-PEG and BP@PDA-PEOz NSs exerted concentration-dependent photothermal effects (Figure 8A). Moreover, over 80% of MCF-7 cells were killed in the presence of 50 μg mL^-1^ BP@PDA-PEOz under 808 nm laser irradiation. Taken together, BP@PDA-PEOz nanocapsule may be an effective PTT agent with desirable biocompatibility.

Furthermore, the BP-DOX@PDA system exhibited pH-dependent cytotoxicity after PEOz modification (Figure 8B). BP-DOX@PDA-PEOz was significantly more toxic at pH 6.8 than at pH 7.4, but BP-DOX@PDA-PEG had almost the same inhibitory effects at pH 7.4 and 6.8, potentially allowing selective killing of cancer cells that were more acidic than normal cells/tissues *in vivo*. Collectively, PEOz modification was conducive to cellular uptake and pH-sensitive drug release in the mildly acidic tumor microenvironment, thereby promoting tumor inhibition and alleviating side effects during chemotherapy.

In addition, MCF-7 cells were treated by DOX, BTZ, DOX + BTZ (1:1) and drug-loaded NSs with the DOX concentrations of 0.25, 1, 2.5, and 5 μg/mL for 24 or 48 h (Figure 8C,D). First, the cytotoxicities of free DOX, BTZ, DTX + BTZ (1:1) and drug-loaded NSs were time- and dose-dependent. Second, compared to individually administered DOX or BTZ, directly co-administering DTX + BTZ was more cytotoxic, as suggested by the raised inhibition rate. Furthermore, the survival rate of cells treated with DOX-loaded BP@PDA-PEOz NSs was apparently lower than that of the DOX-loaded BP@PDA-PEG NSs group after incubation for 24 or 48 h. Therefore, PEOz was more conducive to long-term drug circulation *in vivo* than PEG, extending the half-lives of drugs and enriching pH-sensitive drugs at tumor sites by recognizing the acidic tumor microenvironment. Most importantly, the group treated by DOX-loaded BP@PDA-PEOz-BTZ in combination with 808 nm laser irradiation (1.0 W cm^-2^) had the lowest survival rate after 48 h of incubation, demonstrating that chemotherapy plus PTT exerted the strongest cytotoxic effects. In short, the antitumor effects of drug were effectively boosted with this pH-sensitive release pattern triggered by NIR laser irradiation, accompanied by minimal side effects.

### *In vivo* IR Thermal Images

**FIGURE 9:**
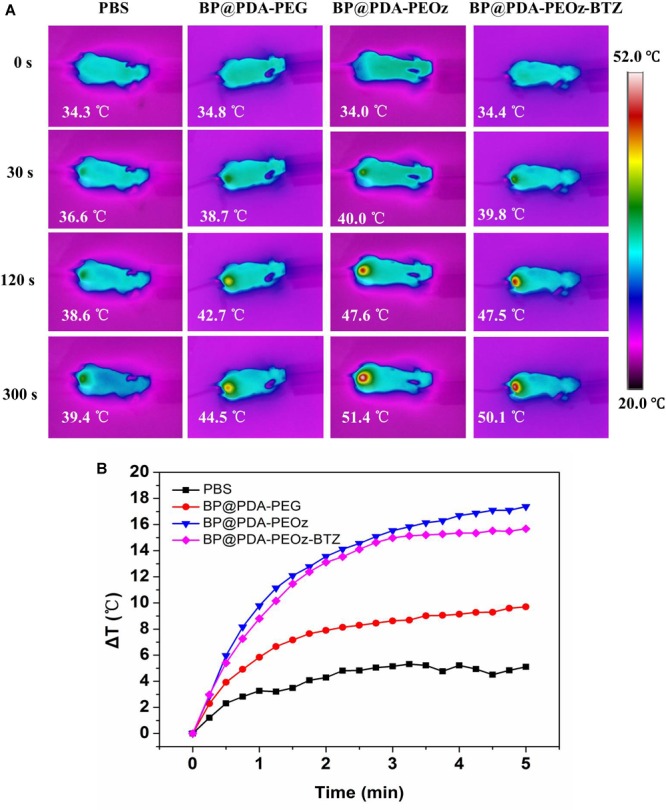
**(A)** *In vivo* IR thermal images of tumor-bearing mice after tail intravenous injection of PBS, BP@PDA-PEG, BP@PDA-PEOz, and BP@PDA-PEOz-BTZ for 24 h, followed by exposure to 808 nm laser irradiation (1.5 W/cm^2^, 5 min). **(B)** Time-dependent temperature increase of MCF-7 tumor-bearing mice recorded by an IR camera under 808 nm laser (1.5 W cm^-2^).

The photothermal efficacy of this BP-based nanoplatform was further studied by acquiring IR thermal images (Figure 9A). 24 h after intravenous injection of BP@PDA-PEG, BP@PDA-PEOz, and BP@PDA-PEOz-BTZ, the tumor sites were irradiated for 5 min by 808 nm laser (1.5 W cm^-2^), and the temperatures in tumor site were measured every 15 s. The tumor surface temperatures significantly increased after NIR irradiation, which can be attributed to the remarkable photothermal effects of PDA coating and BP. The tumor temperatures of BP@PDA-PEOz group and BP@PDA-PEOz-BTZ group rapidly increased to 51.4 and 50.1°C, respectively, which were sufficiently high for effective ablation. The *in vivo* local tumor temperatures of BP@PDA-PEOz and BP@PDA-PEOz-BTZ groups changed more obviously than that of the BP@PDA-PEG group did, because PEOz underwent charge reversal from negative to positive upon tertiary amide group ionization along the PEOz chain in tumor tissue with a low pH, ultimately benefiting the uptake by cancer cells. On the contrary, the temperature of PBS-treated tumor did not rise evidently after irradiation under identical conditions, so cancer cells remained intact. Based on long-term circulation *in vivo*, the PEOz-modified, BP-based NS drug delivery platform was photothermally active, being capable of pH-triggered targeting of tumor tissues. The quantified tumor temperature variations are presented in Figure 9B.

## Conclusion

In summary, we have successfully developed an effective pH-responsive and dual drug co-delivery nanoplatform (DOX-loaded BP@PDA-PEOz-BTZ) for combined chemotherapy and PTT. Not only did we show PDA coating can enhance both biostability and photothermal activity of the BP NS, we also demonstrated that PEOz conjugation can improve the targeted, long circulation *in vivo* as well as pH- and photo-responsive drug release, indicating that PEOz is an excellent substitute for PEG. Given the high drug encapsulation efficiency, the strong cellular uptake and cytotoxicity, together with the photo-responsive, rapid drug release triggered by low pH, our versatile PDA- and PEOz-modified, BP-based dual drug co-delivery nanoplatform has great potentials for synergistic cancer treatment.

## Author Contributions

ZP designed the research project. NG, CX, and LF had full controlled the experiments, data analysis, and preparation of article. HW, XZ, and LM were involved in planning the analysis and drafting the article. The final draft article was approved by all the authors.

## Conflict of Interest Statement

The authors declare that the research was conducted in the absence of any commercial or financial relationships that could be construed as a potential conflict of interest.
