# Supplementary material for: pH-Responsive Dual Drug-Loaded Nanocarriers Based on Poly (2-Ethyl-2-Oxazoline) Modified Black Phosphorus Nanosheets for Cancer Chemo/Photothermal Therapy
Source: Front Pharmacol. 2019 Mar 19;10:270. doi: 10.3389/fphar.2019.00270 (PMC6433829; doi:10.3389/fphar.2019.00270)
Supplement: Supplementary file 1 [file Data_Sheet_1.docx]

***Supplementary Information***

**pH-Responsive Dual Drug-Loaded Nanocarriers Based on Poly (2-Ethyl-2-Oxazoline) Modified Black Phosphorus Nanosheets for Cancer Chemo/Photothermal Therapy**

Nansha Gao^a^, Chenyang Xing^a^, Haifei Wang^a^, Liwen Feng^b^, Xiaowei Zeng^b^, Lin Mei^b^, Zhengchun Peng^a,*^

*^a^ Key Laboratory of Optoelectronic Devices and Systems of Ministry of Education, College of Optoelectronic Engineering, Shenzhen University, Shenzhen 518060, China.*

*^b^ School of Pharmaceutical Sciences (Shenzhen), Sun Yat-sen University, Guangzhou 510275, China*

^*^ Corresponding author

E-mail: zcpeng@szu.edu.cn (Z. Peng)


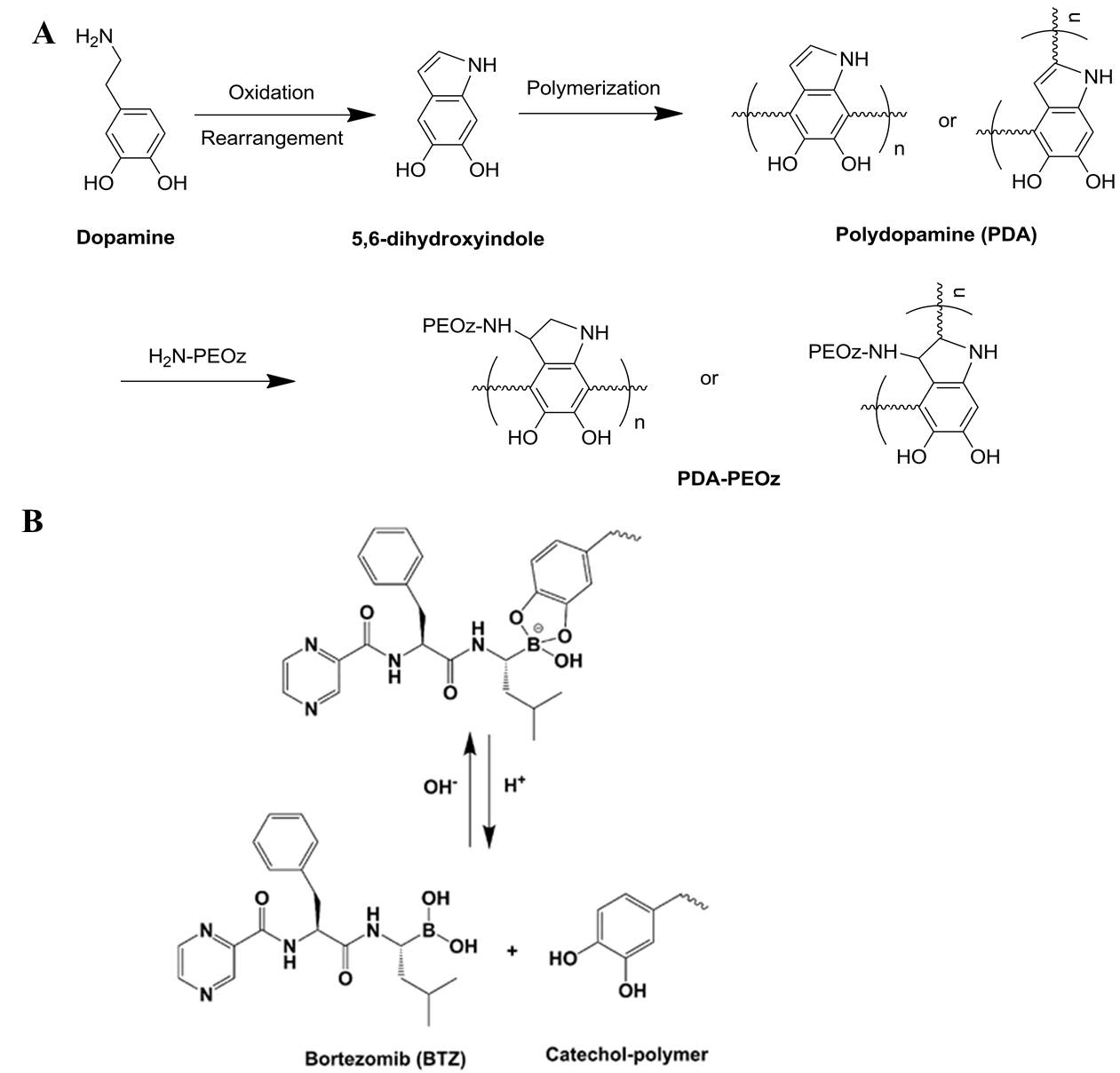


**Figure S1.** (A) The oxidative self-polymerization mechanism of dopamine and the conjugation mechanism between H_2_N-PEOz and PDA coating. (B)The covalent bond of catechol and boronic acid structure in BTZ forms and remains stable at neutral and alkaline pH. However, it dissociates in acid environment easily. The pH-responsive bond contributes to inhibit the activity of BTZ in neutral circulation and release free BTZ at the acid tumor sites.


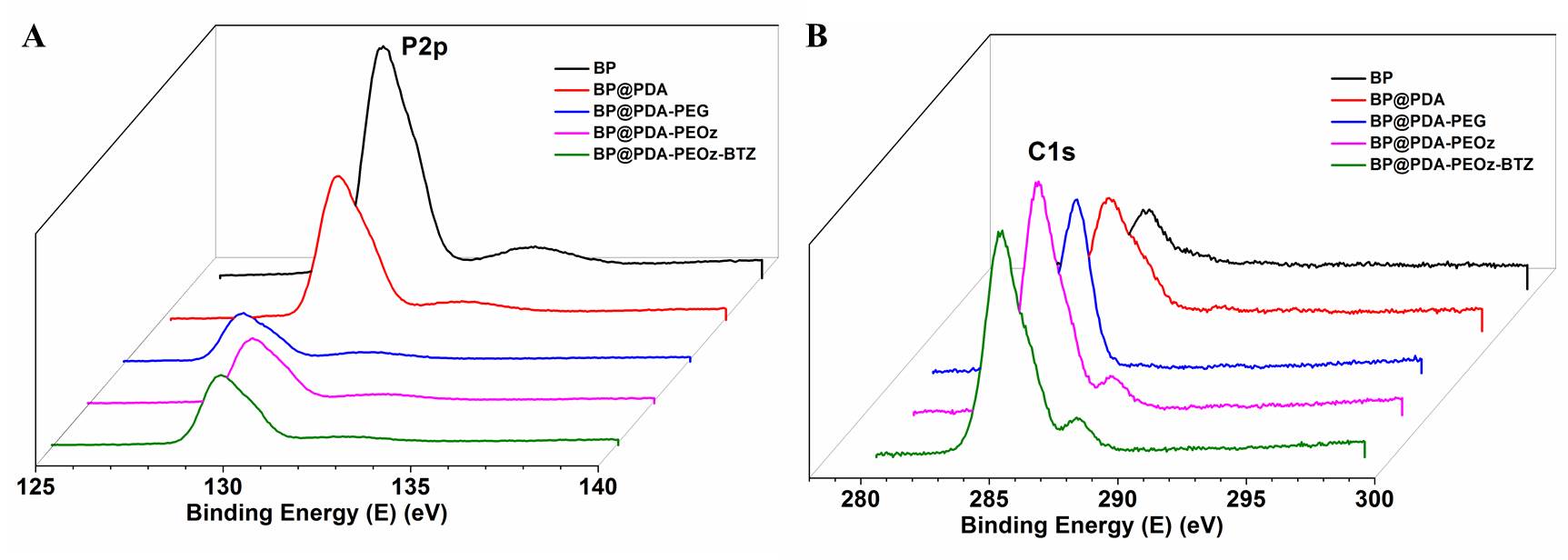


**Figure S2.** XPS spectra of BP, BP@PDA, BP@PDA-PEG, BP@PDA-PEOz, BP@PDA-PEOz-BTZ: (A) P2p spectrum, and (B) C1s spectrum.


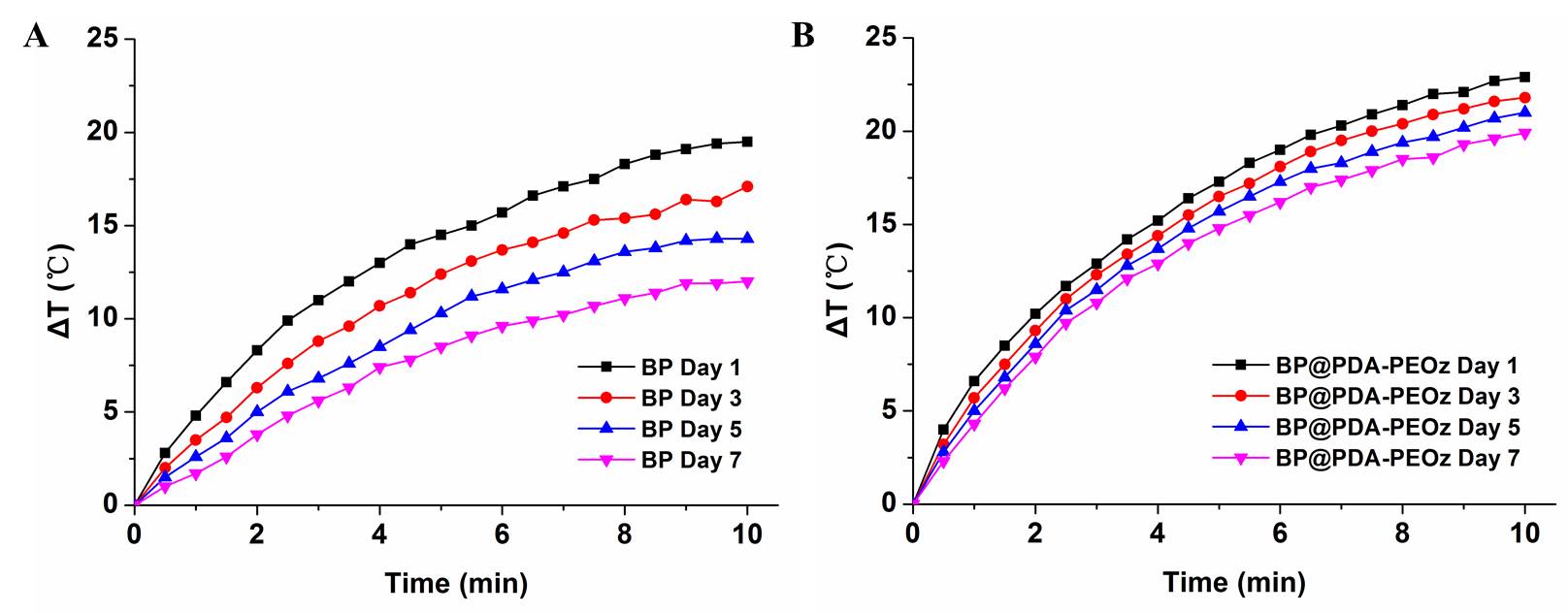


**Figure S3.** Photothermal curves of BP and BP@PDA-PEOz NSs, respectively, after storing in water for different periods of time under 808 nm laser irradiation (1.0 W cm^−2^) for 10 min.


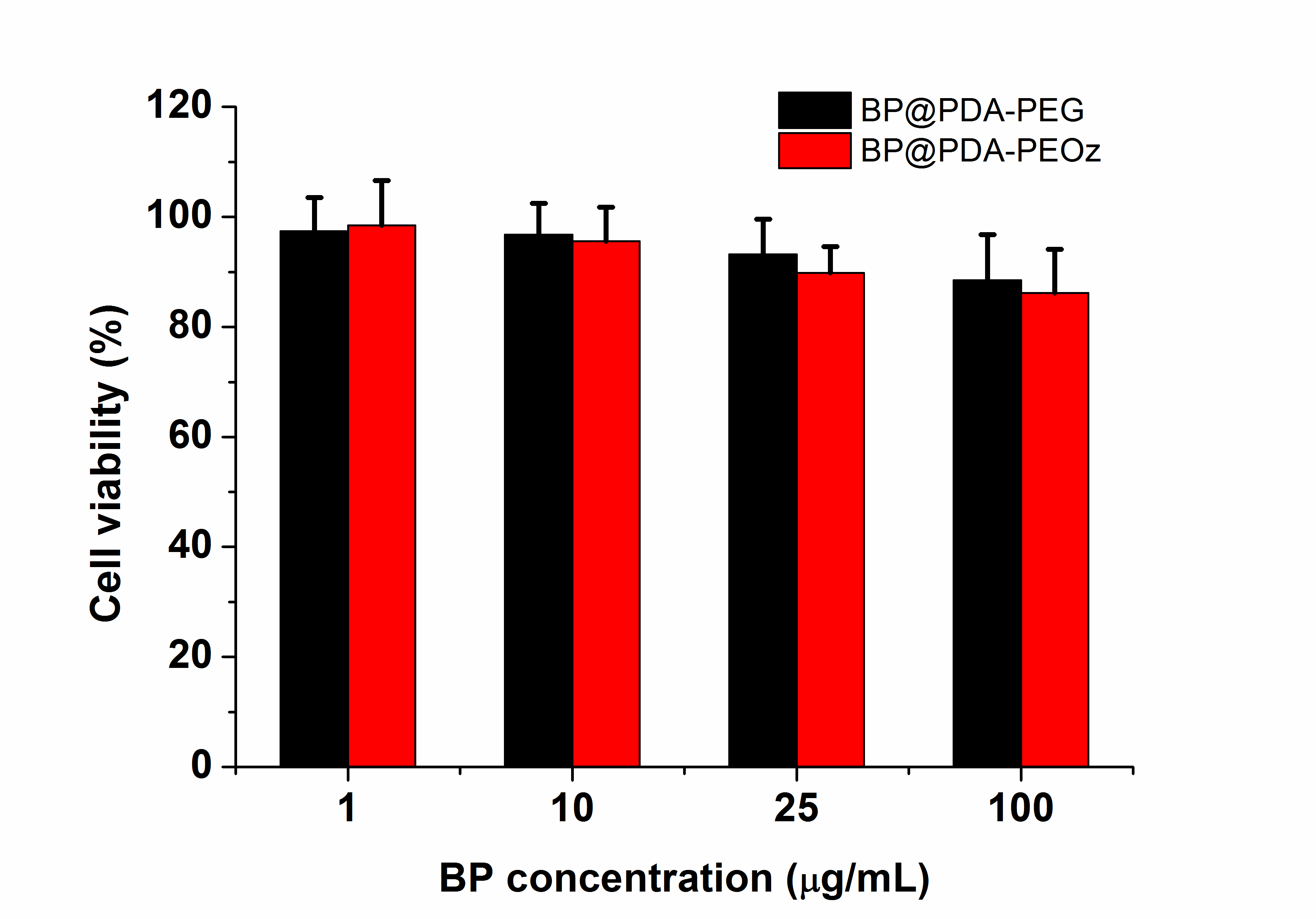


**Figure S4.** Cell viability of MCF-7 cells cultured with drug-free BP@PDA-PEG and BP@PDA-PEOz at a NPs concentration series of 1, 10, 25, and 100 μg/mL for 48 h.
